# Genetic Basis of Gluten Aggregation Properties in Wheat (*Triticum aestivum* L.) Dissected by QTL Mapping of GlutoPeak Parameters

**DOI:** 10.3389/fpls.2020.611605

**Published:** 2021-01-28

**Authors:** Zhengfu Zhou, Ziwei Zhang, Lihua Jia, Hongxia Qiu, Huiyue Guan, Congcong Liu, Maomao Qin, Yahuan Wang, Wenxu Li, Wen Yao, Zhengqing Wu, Baoming Tian, Zhensheng Lei

**Affiliations:** ^1^Henan Institute of Crop Molecular Breeding, Henan Academy of Agricultural Sciences, Zhengzhou, China; ^2^Agronomy College, Zhengzhou University, Zhengzhou, China; ^3^National Key Laboratory of Wheat and Maize Crop Science, Henan Agricultural University, Zhengzhou, China

**Keywords:** wheat, QTL mapping, gluten aggregation properties, GlutoPeak parameters, wheat quality

## Abstract

Bread wheat is one of the most important crops worldwide, supplying approximately one-fifth of the daily protein and the calories for human consumption. Gluten aggregation properties play important roles in determining the processing quality of wheat (*Triticum aestivum* L.) products. Nevertheless, the genetic basis of gluten aggregation properties has not been reported so far. In this study, a recombinant inbred line (RIL) population derived from the cross between Luozhen No. 1 and Zhengyumai 9987 was used to identify quantitative trait loci (QTL) underlying gluten aggregation properties with GlutoPeak parameters. A linkage map was constructed based on 8,518 SNPs genotyped by specific length amplified fragment sequencing (SLAF-seq). A total of 33 additive QTLs on 14 chromosomes were detected by genome-wide composite interval mapping (GCIM), four of which accounted for more than 10% of the phenotypic variation across three environments. Two major QTL clusters were identified on chromosomes 1DS and 1DL. A premature termination of codon (PTC) mutation in the candidate gene (*TraesCS1D02G009900*) of the QTL cluster on 1DS was detected between Luozhen No. 1 and Zhengyumai 9987, which might be responsible for the difference in gluten aggregation properties between the two varieties. Subsequently, two KASP markers were designed based on SNPs in stringent linkage with the two major QTL clusters. Results of this study provide new insights into the genetic architecture of gluten aggregation properties in wheat, which are helpful for future improvement of the processing quality in wheat breeding.

## Introduction

Common wheat is one of the most important crops worldwide, which provides one-fifth of the calories consumed by humans (Dubcovsky and Dvorak, 2007). As the main components of wheat endosperm, proteins play important roles in determining the processing quality of wheat. The functional properties of wheat are largely determined by the composition of gluten proteins and the interactions between different gluten proteins upon addition of water when forming a dough (Shewry and Tatham, 1997; Sliwinski et al., 2004a,b). Gluten proteins consist of glutenin and gliadin, and are responsible for the rheological properties of dough. Glutenin is divided into high- and low-molecular weight glutenin subunits (HMW-GSs and LMW-GSs), encoded by *Glu-1* and *Glu-3* loci on chromosomes 1A, 1B, and 1D, respectively (Payne, 1987). The role of HMW-GSs on dough strength has been well studied (Ram, 2003; Edwards et al., 2007; Zhang et al., 2007), whereas there has been a lack of studies on the regulation of grain quality by LMW-GSs, although LMW-GSs were reported to play significant roles on dough viscosity and formation of large polymers (Cornish et al., 2001). Gliadin is a significant agent regulating viscoelastic properties of gluten (Xu et al., 2007). It is generally agreed that subunits or alleles *Glu-A1a*, *Glu-B1b*, *Glu-D1d*, *Glu-A3b*, *Glu-A3d*, *Glu-B3d*, *Glu-B3g*, *GliB1b*, *GliA2b*, and *GliB2c* confer superior dough rheological properties (Metakovsky et al., 1997; He et al., 2005; Liu et al., 2005; Liang et al., 2010; Jin et al., 2013).

Flour properties are largely determined by the quality of gluten, which are usually assessed by means of classical rheological tests, such as Farinograph, Alveograph, or Extensograph analyses (Sun et al., 2010). Unfortunately, a full characteristic of wheat grain based on classical tests are time and labor consuming (Marti et al., 2015a). Recently, a new device, GlutoPeak, was introduced to assess gluten aggregation properties, and a rapid shear-based method was developed for discriminating the gluten quality (Rakita et al., 2018). In addition, much smaller samples are required by the new method (< 10 g). By mixing appropriate amounts of flour and solvent at a certain speed, gluten is separated by the paddle rotation, resulting in aggregation. At this point the gluten aggregate mass exerts a resistant force on the paddle resulting in the generation of a torque curve. The curve records the complexity of aggregation and breakdown of the gluten by providing peak maximum time (*PMT*), maximum torque (*BEM*), and other parameters (Wang et al., 2018). It has been shown that the parameters *PMT*, torque 15 s before the *BEM* (*AM*), torque 15 s after the *BEM* (*PM*) and area from the first minimum to *AM* (A3) of GlutoPeak were significantly correlated with the test parameters of the Farinograph, Extensograph, and Alveograph (Melnyk et al., 2012; Chandi et al., 2015; Marti et al., 2015b; Zawieja et al., 2020). By analyzing durum wheat semolina utilizing GlutoPeak and the conventional methods, Sissons (2016) found that the parameter *PMT* of GlutoPeak is able to separate weak and strong gluten index samples. Rakita et al. (2018) confirmed that GlutoPeak was successful in discriminating wheat varieties of good quality from those of poor quality. A systematic study was conducted by Wang et al. (2017) to elucidate the impact of water absorption (WA) and gluten strength on gluten aggregation behavior in GlutoPeak tests. These studies suggested that GlutoPeak can be used as a reliable and fast approach to evaluate the quality of wheat gluten.

Dozens of studies were reported to dissect the genetic basis of gluten quality, which were quantitative traits controlled by multiple genes (Branlard et al., 2001; He et al., 2004). Although significantly influenced by environmental variations (Peterson et al., 1992; Bao et al., 2004; Zhang et al., 2005), major QTL for gluten quality have been frequently reported (Eagles et al., 2002; Patil et al., 2009). Patil et al. (2009) identified regions on chromosomes 1A, 4A, 5B, 6A, and 7A underlying gluten quality in addition to the major QTL on chromosome 1B. Conti et al. (2011) identified 11 QTL for gluten quality located on chromosomes 1A, 1B, 3B, 4A, 4B, 6A, 6B, and 7A, explaining 4.9–54.2% of the phenotypic variation. Jin et al. (2016) reported 119 additive QTL for gluten quality on 20 wheat chromosomes, among which 11 QTL clusters were identified for Mixograph, RVA, and Mixolab parameters of gluten quality.

Nevertheless, genetic dissection of gluten quality utilizing GlutoPeak parameters has not been reported so far. The associations of GlutoPeak parameters with gluten aggregation properties and the molecular mechanisms underlying gluten aggregation properties in wheat remain unclear. In this study, we employed a recombinant inbred line (RIL) population derived from the cross between Luozhen No. 1 and Zhengyumai 9987 to dissect QTL for gluten aggregation properties evaluated by GlutoPeak parameters. A linkage map was constructed with the SLAF-seq technique, and 33 QTLs were identified for nine GlutoPeak parameters. A candidate gene for the QTL cluster on chromosome 1DS was identified; we further performed additive effect analysis and KASP marker design of the two QTL clusters. Results of this study provides new insights into the genetic mechanisms for wheat grain quality, which are helpful to future wheat breeding efforts targeting the improvement of gluten aggregation properties of wheat.

## Materials and Methods

### Plant Materials

The F_6_ generation of a RIL population consisting of 196 wheat lines was generated by crossing between Luozhen No. 1 and Zhengyumai 9987, which were used for genetic mapping and QTL discovery. A large amount of data was collected for the two varieties in previous studies of our lab, and significant differences were observed between the two varieties in Farinograph parameters, Extensograph parameters, and other quality-related parameters. The RIL population and the parents were grown under four environments, including Yuanyang 2017–2018 (E113°97′, N35°05′), Yanjin 2017–2018 (E114°20′, N35°14′), Yuanyang 2018–2019 (E113°97′, N35°05′), and Shangqiu 2018–2019 (E115°65′, N34°45′) in northern China. The average temperatures were 14.3°C, 14°C, and 14.2°C, while the average rainfalls were 556, 656, and 623 mm per year, for Yuanyang, Yanjin, and Shangqiu, respectively. An additional 39 wheat cultivars collected from Henan Province were also planted in 2018 in Yuanyang to establish the correlations between the GlutoPeak parameters and gluten parameters measured by traditional methods. Random block design was used to plant wheat accessions according to two rows of plot, three repetitions; each row has a length of 2 m and a width of 23 cm, plant spacing 10 cm. All wheat accessions were cultivated using standard agronomic practices and were harvested at physiological maturity (8–10% moisture content) under each environment to minimize variation across different environments.

### Evaluation of Dough Rheological Parameters

Samples of 39 wheat accessions from Henan Province, China were milled following American Association of Cereal Chemists (AACC) approved method 26–21.02 utilizing Brabender Quadrumat Junior Mill (Brabender, Germany). The moisture and protein content test of flour was conducted based on AACC approved method 46–30.01 utilizing IM9500 (Perten, Sweden). The wet gluten content test of flour was conducted based on AACC approved method 38–12A utilizing GM2200 (Perten, Sweden). The Farinograph test of flour was conducted based on AACC approved method 54–21.02 utilizing Farinograph-E (Brabender, Germany). The Extensograph test of flour was conducted based on AACC approved method 5410 utilizing Extensograph-E (Brabender, Germany). In addition, the whole-wheat meal of 39 samples obtained utilizing LM3100 (Perten, Sweden) were tested by GlutoPeak. Each test was replicated three times, and the average values were used for further analysis.

### Collection of Phenotypic Data

The whole-wheat meal was prepared utilizing LM3100 (Perten, Sweden) and stored at low temperature (4–5°C). Gluten aggregation properties of whole-wheat meal were measured by GlutoPeak (Brabender, Germany) with a high shear under four environments. The sample weight and 0.5 mol/L CaCl_2_ solution weight were calculated based on the moisture of the sample using the software GlutoPeak. The speed of the rotating paddle was set at 1,900 rpm, and the temperature was set at 34°C by circulating water through the jacketed sample cup (Chandi and Seetharaman, 2012). All measurements were performed twice and the average values were used for further analysis.

During the GlutoPeak test, the counter torque generated and gluten network formation upon mixing and the time required to reach peak resistance were recorded in a torque curve (Supplementary Figure 1; Fu et al., 2017). Nine parameters including peak maximum time (*PMT*), maximum torque (*BEM*), torque 15 s before the *BEM* (*AM*), torque 15 s after the *BEM* (*PM*), area from the beginning of the test to the first maximum (A1), area from the first maximum to the first minimum (A2), area from the first minimum to *AM* (A3), area from *AM* to *BEM* (A4), and area from *BEM* to *PM* (A5) (Amoriello et al., 2015) were collected for QTL analysis.

### Statistical Analysis

Frequency distribution map was made using Origin (OriginLab, United States). Descriptive statistics analysis of the phenotypic data from each environment was conducted using the psych package of the R software. The statistical *p* value of normality test for each trait was performed using the Shapiro–Wilk Normality Testing in R software. Student’s *t*-test and analysis of variance (ANOVA) was performed using the international general SAS 9.2 (SAS Institute, United States) statistical software. Best linear unbiased predictor (BLUP) values for each sample across the four environments and broad-sense heritability of each parameter were obtained by fitting the mixed linear model using the “lme4” package of the R software (R Development Core Team, 2012). Correlation analysis between different GlutoPeak parameters was performed in the R software using the BLUP values.

### Linkage Map Construction and QTL Mapping

The linkage map of the RIL population was constructed by the High Map software based on SNPs obtained utilizing the SLAF-seq technique of Biomarker Biotech Co., Ltd. QTL analysis was performed using the “QTL.gCIMapping.GUI” (Quantitative Trait Loci Genome-Wide Composite Interval Mapping with Graphical User Interface^1^) R software package. Significant QTL were determined by a LOD value threshold of 2.5. The software Mapchart 2.3 was used to map the position of QTL on wheat chromosomes.

### Prediction of Candidate Genes

The physical positions of all significant QTL were determined by aligning sequences of flanking markers against IWGSC (International Wheat Genome Sequence Consortium) RefSeq v1.1 genome^2^ via BLAST. Annotations of protein-coding genes in the QTL interval were extracted from the high confidence wheat gene list provided by IWGSC. The expression profiles of candidate genes were obtained from the public database of wheat expression browser^3^. The heat map was drawn using TBtools. Candidate genes were predicted based on the annotation results and expression profiles of genes in the confidence interval of QTL clusters.

### Molecular Cloning and Sequence Analysis of Candidate Genes

The sequences of candidate genes were obtained based on the Chinese spring (CS) genome at EnsemblPlants^4^. Primers were designed based on conserved sequences flanking candidate genes utilizing the Oligo 7 software and were further synthesized by Sangon Biotech Co., Ltd. The PCR products were separated by electrophoresis on 1% agarose gels, which were cleaned and cloned into the pEASY-Blunt3 vector (Beijing TransGen Biotech Co., Ltd.^5^). The vector was then transformed into DH5α competent *E. coli* cells by the heat shock method. Single clones were sequenced by Sangon Biotech Co., Ltd., and sequence analysis (alignment and assembly) was performed using the DNAMAN software^6^ and the GENEDOC software. According to the method used by DuPont et al. (2005), whole wheat flour was used to extract gluten, and RP-HPLC was used to determine gluten and its subunit components (González-Torralba et al., 2011).

KASP primers were designed based on the differences from the cloned sequences between the RIL parents, which were then used to screen all 196 RILs. The JoinMap software was used to map the gene onto the genetic map based on the genotypes of these marker and neighboring markers. The SNP marker and neighboring markers were evaluated by their physical order on the Chinese Spring reference genome (IWGSC RefSeq v1.1^2^) to confirm the correct location of cloned genes.

### Development of Gene-Specific KASP Markers

KASP markers were designed for the two major QTL clusters based on single-nucleotide polymorphisms. Each 10 μl of PCR mixture for the KASP genotyping procedure contained 100 ng of template DNA, 5 μl of KASP master mix, 4 mM MgCl_2_, and 1.4 μl of primer mixture, which comprised 46 μl of ddH_2_O, 30 μl of common primer (10 μM), and 12 μl of each tailed primer (10 μM). PCR cycling was performed using the following protocol: a hot start at 95°C for 15 min, 10 touchdown cycles (95°C for 20 s; touchdown at 65°C initially and decreasing by −1°C per cycle for 60 s), and 30 additional cycles of annealing (95°C for 10 s; 57°C for 60 s). Then, an extension step (each three cycles of annealing: 95°C for 10 s; 57°C for 60 s) was replicated three times to obtain the best genotyping cluster result (Rasheed et al., 2016). A final 37°C step was used to get the genotyping results. The primers were synthetized by Sangon Biotech (Shanghai, China).

## Results

### Correlations Between GlutoPeak Parameters and Dough Rheological Parameters

We performed correlation analysis between the GlutoPeak parameters and the dough rheological parameters utilizing the samples of 39 wheat accessions (see section “MATERIALS AND METHODS”) (Table 1; Supplementary Table 1). Almost all GlutoPeak parameters were significantly correlated with the dough rheological parameters, with correlation coefficients ranging from 0.28 to 0.91. Especially, the *PMT*, *AM*, area from the beginning of the test to the first maximum (A1) and A3 values (GlutoPeak parameters) were significantly positively correlated with the dough stabilization time (DST), dough maximum resistance to extension (*R*max), and extensigram area (EA) values (dough rheological parameters), with correlation coefficients ranging from 0.71 to 0.91.

**TABLE 1:** Correlation coefficients (*r*) between GlutoPeak parameters of whole-wheat meal and dough rheological parameters of flour.

GlutoPeak parameters	Dough rheological parameters					
	
	GPC	WGC	WA	DST	*R*max	EA
*PMT*	0.60**	0.44**	0.19^*ns*^	0.76**	0.90**	0.89**
*BEM*	0.68**	0.68**	0.35*	0.38**	0.51**	0.59**
*AM*	0.71**	0.57**	0.38**	0.75**	0.87**	0.89**
*PM*	0.81**	0.69**	0.68**	0.76**	0.80**	0.83**
A1	0.73**	0.64**	0.28*	0.71**	0.89**	0.90**
A2	0.55**	0.58**	0.13^*ns*^	0.37*	0.62**	0.60**
A3	0.65**	0.54**	0.22^*ns*^	0.72**	0.91**	0.89**
A4	0.77**	0.76**	0.39**	0.58**	0.74**	0.80**
A5	0.74**	0.66**	0.50**	0.61**	0.68**	0.74**

GPC, grain protein content; WGC, wet gluten content; WA, water absorption; DST, dough stabilization time; *R*max, dough maximum resistance to extension; EA, extensigram area; PMT, peak maximum time; BEM, maximum torque; AM, torque 15 s before the BEM; PM, torque 15 s after the BEM; A1, area from the beginning of the test to the first maximum; A2, area from the first maximum to the first minimum; A3, area from the first minimum to AM; A4, area from AM to BEM; A5, area from BEM to PM. *, *p* < 0.05. **, *p* < 0.01. ns, no significant difference.

Based on the results of correlation analysis, the *PMT*, *AM*, A1, and A3 values of whole-wheat meal were found to be good indicators of wheat flour quality and extension parameters. These parameters can be used to establish the rheological properties of wheat flour dough, which can also be used to estimate the qualities of wheat grains measured by Farinograph and Extensograph.

### Phenotypic Investigation of the RIL Population

**FIGURE 1:**
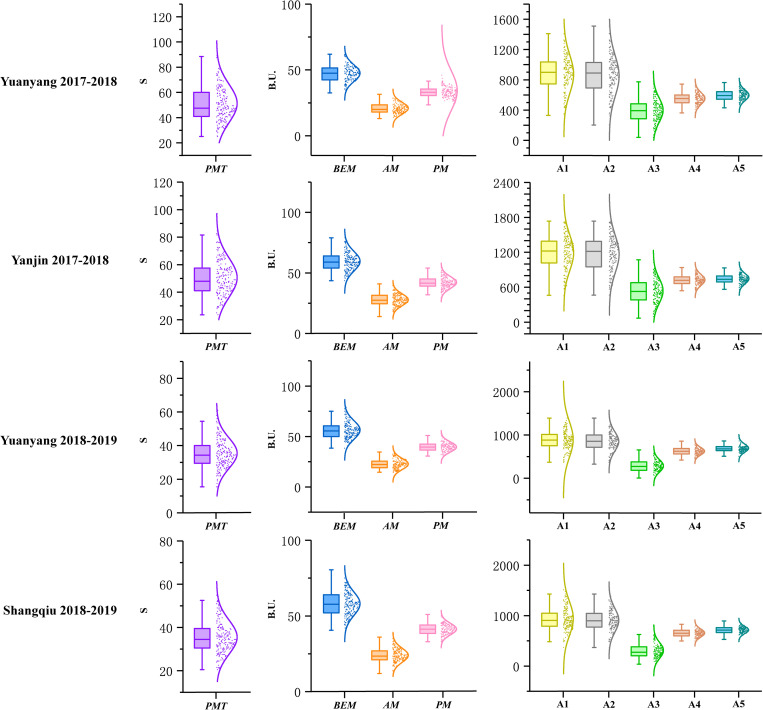
Distributions of the parameters tested by GlutoPeak for the recombinant inbred line (RIL) population derived from the cross between Luozhen No. 1 and Zhengyumai 9987 under four environments. See footnote of Table 1 for abbreviations. The raw data values are displayed as scatter points, while the probability density of the data is demonstrated as a smooth curve. The horizontal line inside the boxplot represents the median value, while the lower and upper borders of the box indicate the lower and upper quartile of the data value. The whiskers of the boxplot define the limits of the data values.

A total of 196 recombinant inbred lines (RILs) derived from the cross between Luozhen No. 1 and Zhengyumai 9987 were evaluated for parameter values including *PMT*, *BEM*, *AM*, *PM*, A1, A2, A3, A4, and A5 using GlutoPeak under four environments (Supplementary Table 2). The value of nine GlutoPeak parameters for Luozhen No. 1 was higher than that of Zhengyumai 9987 under each environment (Supplementary Table 3). Significant differences of these parameters were observed between the two parents (Supplementary Figure 2). Most of the parameters were nearly normally distributed as shown in Figure 1 (Supplementary Table 4).

The contributions of variance in genotype (G), environment (E) and G × E interaction were assessed by ANOVA. Significant effects on all parameters were observed for genotype variations. For environmental variations, significant effects were observed on all parameters. The broad-sense heritability for different parameters ranged from 0.43 to 0.83 (Supplementary Table 5).

Significant positive correlations were found among *PMT*, *AM*, A1, A2, and A3 while significant negative correlations were found among *PMT*, *BEM*, *PM*, and A5 (Supplementary Figure 3). *PMT* and A3 were the most vital parameters regarding aggregation properties of gluten, which were highly positively correlated with each other (*r* = 0.93).

### Construction of a Linkage Map for the Recombinant Inbrid Line Population

A genetic map was constructed for the RIL population, which consisted of 8,518 SNPs distributed across all 21 wheat chromosomes and covered 3,140.54 cM (see section “MATERIALS AND METHODS”) (Supplementary Table 6). The length of different chromosomes ranged from 94.33 cM (3D) to 207.17 cM (7A), with an average length of 149.55 cM. More than 98.76% of the distances between adjacent SNPs were smaller than 5 cM, while the max distance between adjacent SNPs was 17.39 cM. A total of 3,415 (40.1%), 3,854 (45.2%), and 1,249 SNPs (14.7%) were distributed in the A, B, and D genomes, representing marker densities of 0.35, 0.30, and 0.64 SNPs/cM, respectively. The number of SNP markers in each chromosome ranged from 106 (7D) to 996 (7A) (Supplementary Table 7).

### QTL Analysis of GlutoPeak Parameters Using the RIL Population

A total of 33 additive QTLs were identified for nine GlutoPeak parameters using the linkage map (Supplementary Table 8). Among all QTLs, the trait-increasing allele of 14 QTLs was derived from Luozhen No. 1, while the trait-increasing allele of 19 QTLs was derived from Zhengyumai 9987. A total of 7, 8, 11, 4, 5, 4, 6, 9, and 11 QTL were identified for *PMT*, *BEM*, *AM*, *PM*, A1, A2, A3, A4, and A5, respectively. *QA2.1DS.1*, *QA3.1DL.2*, *QA4.3AL*, and *QA5.3AS.1* explained more than 10% of the phenotypic variation of A2, A3, A4, and A5, separately. *QA3.1DL.2* explained the highest phenotypic variation (20.19% to 43.50%) of all QTLs. Many QTLs detected under four environments, and the BLUP values of both environments were colocalized with each other, including *QPMT.1DL.2*, *QA3.1DS.1*, and *QA3.1DL.2*. (Supplementary Figure 5 and Supplementary Table 8).

### QTL Clusters for GlutoPeak Parameters

**FIGURE 2:**
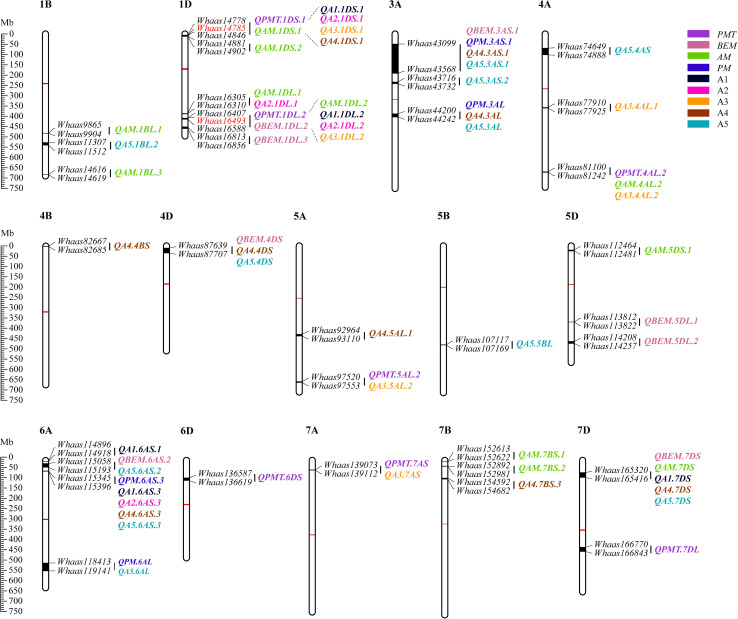
Physical maps and quantitative trait loci (QTL) for Glutopeak parameters identified using the Luozhen No. 1/Zhengyumai 9987 population. The centromere and the QTL intervals on each chromosome are indicated by red and black rectangles, respectively. QTL for different parameters defined in Table 1 are represented by different colors. The SNPs used for KASP marker development are marked in red color. See footnote of Table 1 for abbreviations.

**FIGURE 3:**
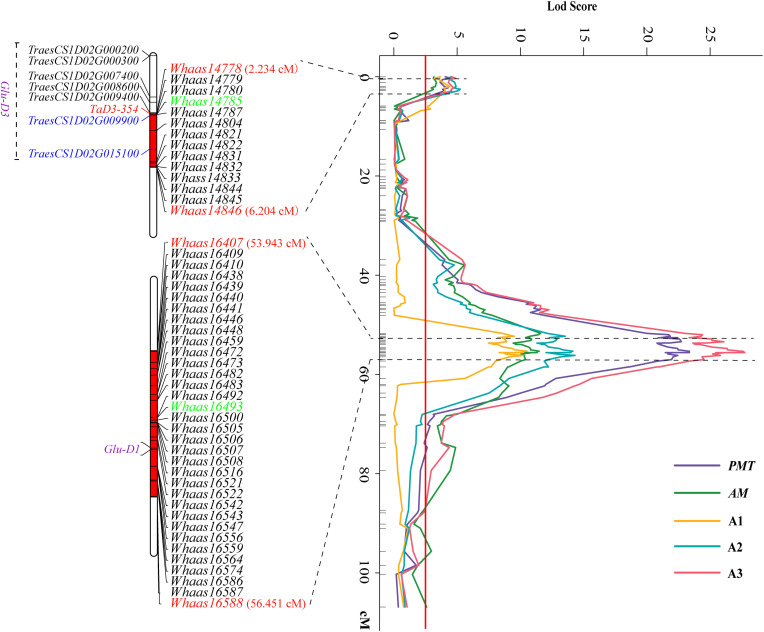
Two major QTL clusters on chromosome 1D underlying gluten aggregation properties. Colored lines represent the LOD score of QTL mapping using best linear unbiased predictor (BLUP) values of different parameters tested by GlutoPeak. The LOD score threshold is indicated by the red vertical line. The two QTL clusters are indicated by dashed lines. The SNPs used for KASP marker development are marked in green. See footnote of Table 1 for abbreviations.

Some QTLs for different GlutoPeak parameters were mapped to the same or neighboring marker intervals, probably caused by pleiotropic effects of a single gene or joint effects of tightly linked genes. The genetic positions of molecular markers were converted into physical positions of the wheat reference genome (Chinese Spring V1.1) to compare the genomic positions of different QTL. As a result, eight QTL clusters were identified on chromosomes 1DS, 1DL, 3AS, 3AL, 4AL, 4DS, 6AS, and 7DS (Table 2 and Figure 2). Two QTL clusters on chromosomes 1DS and 1DL were identified as the most prominent QTL clusters for gluten aggregation properties (Figure 3).

**TABLE 2:** QTL clusters detected for GlutoPeak parameters with a recombinant inbred line (RIL) population derived from the cross between Luozhen No. 1 and Zhengyumai 9987.

QTL cluster	Flanking interval	Genetic distance (cM)	Physical position (Mb)	Parameters
1DS.1	*Whaas14778–Whaas14846*	2.2–6.2	5.2–8.3	*PMT* (I, IV, B); *AM* (II, IV, B);
				A1 (I, II, B); A2 (I, IV, B);
				A3 (I, II, III, IV, B); A4 (II, IV).
1DL.2	*Whaas16407–Whaas16588*	53.9–56.5	409.5–414.7	*PMT* (I, II, III, IV, B); *BEM* (B);
				*AM* (I, II, IV, B); A1 (I, II, B);
				A2 (I, II, IV, B); A3 (I, II, III, IV, B).
3AS.1	*Whaas43099–Whaas43568*	36.2–41.8	48.4–191.1	*BEM* (III, IV, B); *PM* (III, IV);
				A4 (III, B); A5 (III, B).
3AL	*Whaas44200–Whaas44242*	44.6–45.1	387.5–403.7	*PM* (II); A4 (II); A5 (II).
4AL.2	*Whaas81100–Whaas81242*	114.5–116.4	669.0–672.0	*PMT* (IV); *AM* (IV, B); A3 (II, IV, B).
4DS	*Whaas87639–Whaas87707*	10.5–24.6	10.2–34.5	*BEM* (I, IV, B); A4 (I); A5 (I, IV, B).
6AS.3	*Whaas115345–Whaas115396*	72.0–75.7	50.4–52.6	*PM* (IV); A1 (II); A2 (B); A4 (IV); A5 (IV).
7DS	*Whaas165320–Whaas165416*	42.2–49.9	59.3–83.3	*BEM* (III, IV, B); *AM* (III); A1 (II);
				A4 (IV, B); A5 (IV, B).

*I, Yuanyang 2017–2018; II, Yanjin 2017–2018; III, Yuanyang 2018–2019; IV, Shangqiu 2018–2019; B, best linear unbiased predictor (BLUP) values for four environments. See footnote of Table 1 for abbreviations.*

The QTL cluster 1DS.1 was anchored between SNP markers *Whaas14778* and *Whaas14846*, corresponding to 1D (5.2–8.3 Mb) of the Chinese Spring reference genome. The QTL cluster 1DL.2 was anchored between SNP markers *Whaas16407* and *Whaas16588*, corresponding to 1D (408.6–414.7 Mb) of the Chinese Spring reference genome. The LOD values of the QTL clusters 1DS.1 and 1DL.2 ranged from 2.69 to 12.12 and 3.27 to 31.82, respectively. *Glu-D1*, the most important locus underlying gluten aggregation properties, overlapped with QTL cluster 1DL.2. We then designed a KASP marker based on SNP *Whaas16493*, which was located in the QTL cluster on 1DL.2 (Supplementary Table 15). The whole RIL population was genotyped using *Whaas16493* and two published KASP markers for *1Dx* and *1Dy* subunit genes of *Glu-D1* locus (Ravel et al., 2020). The KASP markers for *1Dx* and *1Dy* co-segregated with each other completely, whereas recombination was detected in two RILs between the KASP marker of *Whaas16493* and the KASP markers of *1Dx*/*1Dy* subunits of *Glu-D1* (Supplementary Table 9). As a result, we considered *Glu-D1* as the most likely candidate gene for *1DL.2*. Meanwhile, *Glu-D3* was found in the QTL cluster 1DS.1, which was subjected to further analysis in this study.

### Prediction of Candidate Genes for the QTL Cluster on 1DS

The wheat reference genome sequence (IWGSC RefSeq v1.1) was employed to identify the candidate genes accounting for the QTL cluster on 1DS. A total of 110 high-confidence genes were in the QTL cluster on chromosome 1DS flanked by *Whaas14778* and *Whaas14846*. Seven of the 110 genes (*TraesCS1D02G009900*, *TraesCS1D02G011500*, *TraesCS1D02G012000*, *TraesCS1D02G 012100*, *TraesCS1D02G013200*, *TraesCS1D02G015100*, and *TraesCS1D02G018300*) were found to be highly expressed in wheat grains based on expression data obtained from the public database of wheat expression browser^3^ (Supplementary Figure 5 and Supplementary Table 10). *TraesCS1D02G009900* and *TraesCS1D02G015100* were annotated as genes encoding LMW-GSs based on the genome annotation of the Chinese Spring reference genome (Supplementary Table 11). As a result, they were speculated as candidate genes underlying gluten aggregation properties.

### Molecular Cloning and Sequence Analysis of Candidate Genes

**FIGURE 4:**
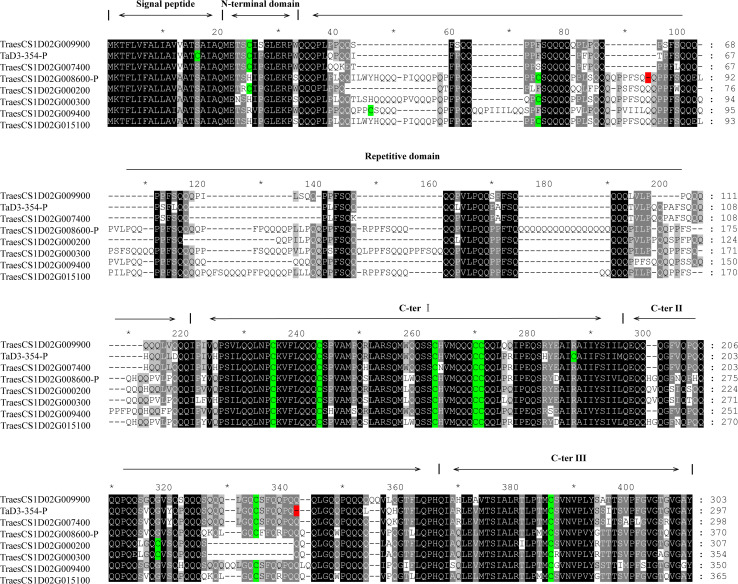
Alignment of putative protein sequences of polymeric protein components of wheat endosperm (LMW-GS) in the major QTL cluster 1DS.1. Cysteine residues are indicated by a green color, while premature termination of codons (PTCs) is indicated by a red color.

The sequence of *TraesCS1D02G009900* was obtained from Ensembl Plants^7^. Primers were then designed based on the conserved sequence flanking this gene in the reference genome to clone the genic sequence of *TraesCS1D02G009900* in Luozhen No. 1 and Zhengyumai 9987 (Supplementary Table 12). An 897-bp sequence was amplified from Luozhen No. 1, while a 912-bp sequence was amplified from Zhengyumai 9987 (Supplementary Table 13). TraesCS1D02G009900 was identified as a typical m-type LMW-GS, containing eight cysteines in the C-terminal domain based on multiple sequence alignment. The amplified sequence from Luozhen No.1 was designated as *TaD3-354*. Seven m-type LMW-GS genes (*TraesCS1D02G000200*, *TraesCS1D02G000300*, *TraesCS1D02G007400*, *TraesCS1D02G008600*, *TraesCS1D02 G009400*, *TraesCS1D02G009900*, and *TraesCS1D02G015100)* were identified at the *Glu-D3* locus of the Chinese Spring reference genome. A PTC mutation at the 234th amino acid position in the C-terminal was detected in TaD3-354 compared with the sequences in Zhengyumai 9987 and the Chinese Spring reference genome (Figure 4). The sequences in Zhengyumai 9987 were identical to the sequence of *TraesCS1D02G009900* in the Chinese Spring reference genome. Moreover, a PTC mutation was identified in the repetitive domain of TraesCS1D02G008600. Then, we developed KASP molecular markers based on the SNPs between *TaD3-354* and *TraesCS1D02G009900* (Supplementary Table 14), which were used in genotyping the RIL population. *TaD3-354* was anchored between *Whaas14779*∼*Whaas14780* on the genetic map, within the interval of the QTL cluster on chromosome 1DS.1 (Figure 3). We further found that the contents of HMW-GSs and LMW-GSs varied between Luozhen No. 1 and Zhengyumai 9987 based on RP-HPLC analysis, indicating that the variation of gluten aggregation properties probably resulted from differentiations in LMW-GSs (Supplementary Figure 6).

### Additive Effects of the Two QTL Clusters

**FIGURE 5:**
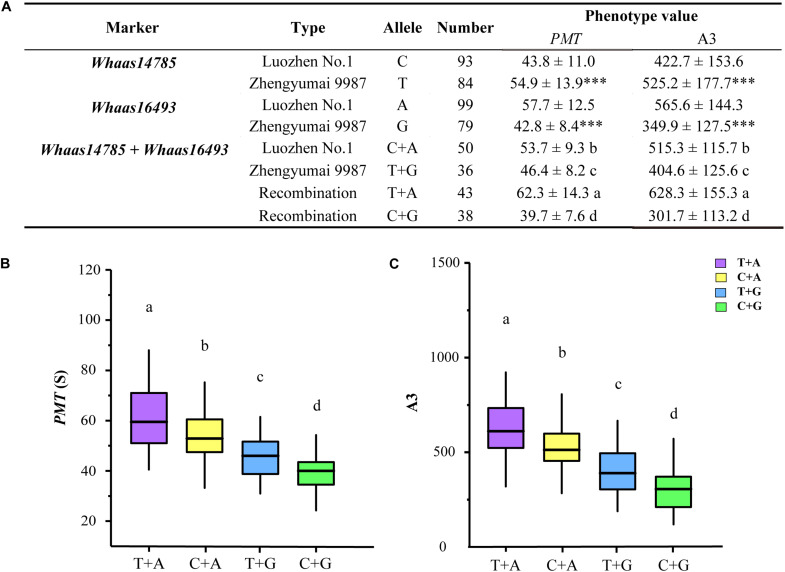
Additive effect analysis of the significant SNPs on chromosome 1D associated with gluten aggregation properties. **(A)** Analysis of variations with different alleles. ^∗∗∗^ *p* < 0.001. **(B)** Comparisons of parameter peak maximum time (*PMT*) among RILs of different genotypes. **(C)** Comparisons of parameter area from the first minimum to *AM* (A3) among RILs of different genotypes. Lowercase letters (a, b, c, d) represent significant differences determined by ANOVA (*p* < 0.001). See footnote of Table 1 for abbreviations.

Based on the genotypes of the RIL population at the two KASP markers representing the two QTL clusters (Supplementary Figure 7 and Supplementary Tables 15, 16), we compared the GlutoPeak parameters (*PMT* and A3) of RILs with different genotypes (Figure 5). At *Whaas14785*, the values of *PMT* and A3 for RILs of Zhengyumai 9987 genotype (T) were higher than that of Luozhen No. 1 (C). On the contrary, the values of *PMT* and A3 for RILs of Zhengyumai 9987 genotype (G) at *Whaas16493* were lower than that of Luozhen No.1 (A). Taking the two QTL clusters together, the phenotype values of RILs with superior allele combination (A + T) were the highest, followed by the phenotype values of RILs with intermediate (A + C, G + T) and inferior (G + C) allele combinations. These results indicated that pyramiding of superior alleles at the two QTL clusters is promising in future wheat breeding.

## Discussion

In this study, we measured the gluten aggregation properties of 39 wheat samples by GlutoPeak, and the dough rheological parameters by Farinograph and Extensograph. We found that the gluten aggregation properties of whole-wheat meal samples were in significant correlation with the rheological parameters. In particular, the parameters *PMT*, *AM*, A1, and A3 of GlutoPeak were highly positively correlated with the dough rheological parameters DST, *R*max, and EA determined by Farinograph and Extensograph (Table 1). As a result, the parameters of Glutopeak can be used to establish the rheological properties of wheat flour dough, and to estimate the qualities of wheat grains measured by Farinograph and Extensograph. Compared with traditional methods to determine wheat quality, the method of GlutoPeak does not require wheat sample moisture adjustment, experimental milling, and post-mature wheat flour sample stabilization treatment, which is much time and labor saving. Our results further confirmed the value of GlutoPeak in researches and breeding targeting wheat grain quality.

Quantitative trait loci (QTL) associated with gluten quality have been detected using SDS sedimentation volume (SV), mixograph parameters, grain protein content (GPC), and gluten index (GI). QTL for gluten quality have been identified on chromosomes 1A, 1B, 1D, 3B, 6A, 6B, 7B, and 7A, and major QTLs for gluten quality were mapped on the long arms of chromosomes 1A, 1B, and 1D, around *Glu-A1*, *Glu-B1*, and *Glu-D1*, respectively (Kunert et al., 2007; Sun et al., 2008; Patil et al., 2009; Conti et al., 2011; Deng et al., 2013, 2015; Kumar et al., 2013; Liu et al., 2017). Nevertheless, QTL analysis of gluten aggregation properties using GlutoPeak parameters has not been conducted up to now. In our study, a total of 33 additive QTL on chromosomes 1B, 1D, 3A, 4A, 4B, 4D, 5A, 5B, 5D, 6A, 6D, 7A, 7B, and 7D were identified using GlutoPeak parameters. Two major QTL clusters were identified on chromosomes 1DS and 1DL for GlutoPeak parameters *PMT*, *AM*, A1, A2, and A3 (Figures 2, 3). The QTL cluster 1DL.2 accounted for 4.70–43.50% of the phenotypic variation and was overlapped with the most important locus (*Glu-D1*) reported to regulate gluten aggregation properties. The QTL cluster 1DS.1 accounted for 3.85–14.23% of the phenotypic variation and was overlapped with another important locus (*Glu-D3*) reported to be responsible for gluten aggregation properties.

By comparing the QTL mapping results with QTLs reported in previous studies, we found that several QTLs were commonly detected in this study and previous studies (Table 3). The QTL cluster of 1DL.2 overlapped with a detected QTL for parameters Mixolab protein weakening torque (C2), Mixolab development time (DT), Mixograph midline peak time (MPT), Mixograph midline peak value (MPV), Mixograph midline 8 min band width (MTxW), and Mixolab stability time (ST) reported by Jin et al. (2016), which also overlapped with a detected QTL for parameters dough development time of Farinograph, dough stabilization time of Farinograph and sedimentation volume reported by Guo et al. (2020). The QTL cluster of 3AS.1 colocalized with a detected QTL regulating grain protein content under late sowing time (LGPC) and wet gluten content under late sowing time (LWGC) reported by Lou et al. (2020). The QTL cluster of 3AL in this study overlapped with a detected QTL for LWGC and grain starch content under normal sowing time (Lou et al., 2020), whereas the QTL cluster of 6AS.3 in this study colocalized with a QTL for parameters ST and SV reported by Guo et al. (2020). On the contrary, the other four QTL clusters of 1DS.1, 4AL.2, 4DS, and 7DS were not reported in previous studies, which were probable novel QTL clusters regulating the characteristics of gluten aggregation. These results suggested the feasibility to dissect the genetic regulation of gluten aggregation properties using GlutoPeak.

**TABLE 3:** Common quantitative trait loci (QTL) clusters detected in this study and previous studies.

QTL cluster	Flanking markers of the QTL	Phenotypic traits regulated by the QTL	References
1DL.2	*Whaas16407*–*Whaas16588*	C2, DT, MPT, MPV, MTxW, ST	Jin et al., 2016
		DDT, DST, SV	Guo et al., 2020
3AS.1	*Whaas43099*–*Whaas43568*	LGPC, LWGC	Lou et al., 2020
3AL	*Whaas44200–Whaas4424*2	LWGC, NGSC	Lou et al., 2020
6AS.3	*Whaas115345*–*Whaas115396*	DST, SV	Guo et al., 2020

*C2, Mixolab protein weakening torque; DT, Mixolab development time; MPT, Mixograph midline peak time; MPV, Mixograph midline peak value; MTxW, Mixograph midline 8 min band width; ST, Mixolab stability time; DDT, dough development time of Farinograph; DST, dough stabilization time of Farinograph; SV, SDS sedimentation volume; LGPC, grain protein content under late sowing time; LWGC, wet gluten content under late sowing time; NGSC, grain starch content under normal sowing time.*

Low molecular weight glutenin subunits are polymeric protein components of wheat endosperm, which are among the biggest macromolecules present in nature, and determine the processing properties of wheat dough (Wei-fu et al., 2018). Based on annotations of the Chinese Spring reference genome and gene expression profiles obtained from the wheat expression browser, we found two LMW-GS genes (*TraesCS1D02G009900* and *TraesCS1D02G015100*) in the QTL cluster on 1DS were highly expressed in wheat grains (Supplementary Figure 5). The two genes were designated as candidate genes for the QTL cluster 1DS.1. We further cloned an LMW-GS gene from the parents of the RIL population, Luozhen No. 1 and Zhengyumai 9987, which was designated as *TaD3-354*, based on the conserved sequences flanking *TraesCS1D02G009900*. A premature termination codon (PTC) mutation in *TaD3-354* was detected between Luozhen No. 1 and Zhengyumai 9987 (Figure 4), which were reported as an important cause for the silence of high-molecular-weight glutenin subunits in *Triticum* species (Chen et al., 2018). *TaD3-354* was anchored in the QTL cluster on chromosome 1DS by genotyping the RIL population with KASP markers designed based on the SNPs in *TaD3-354* and *TraesCS1D02G009900*. We further found that the content of LMW-GSs differed significantly between Luozhen No. 1 and Zhengyumai 9987 through RP-HPLC analysis (Supplementary Figure 6). The results implied that the mutations in LMW-GS gene *TaD3-354* were probably responsible for the difference in gluten aggregation properties between Luozhen No. 1 and Zhengyumai 9987.

Based on the genotypes of the RIL population at the two KASP markers representing the two QTL clusters (Supplementary Figure 7), we found that RILs with different genotypes at the two QTL clusters exhibited significant differences regarding gluten aggregation properties. In addition, we observed significant additive effect of the two QTL clusters on gluten aggregation properties. The phenotype values of RILs with superior allele combination were the highest, followed by the phenotype values of RILs with intermediate and inferior allele combinations (Figure 5). These results indicate that pyramiding of beneficial alleles is a promising approach in wheat breeding targeting gluten aggregation properties. The KASP markers designed in this study are useful in future research efforts and breeding programs on gluten aggregation properties.

## Data Availability Statement

The raw SLAF sequencing data of our manuscript have been uploaded to the public database, which can be found in Genome Sequence Archive (https://bigd.big.ac.cn/gsa/browse/CRA003543).

## Author Contributions

ZFZ, ZWZ, BT, and ZL conceived and designed the experiments. ZWZ, LJ, and HQ carried out phenotype experiments. LJ and HG were responsible for DNA marker analysis. ZWZ, CL, MQ, and WY performed genetic mapping, QTL analysis, and candidate gene identification. YW and WL constructed the mapping population. ZFZ and ZWZ wrote the manuscript. ZL and WY revised the manuscript. All authors read and approved the final manuscript.

## Conflict of Interest

The authors declare that the research was conducted in the absence of any commercial or financial relationships that could be construed as a potential conflict of interest.

## Footnotes

1 https://cran.r-project.org/web/packages/QTL.gCIMapping.GUI/index.html

2 http://www.wheatgenome.org/

3 http://www.wheat-expression.com

4 http://plants.ensembl.org/Triticum_aestivum/Info/Index

5 http://www.transgen.com.cn

6 http://www.lynnon.com

7 http://plants.ensembl.org/index.html
