# Supplementary material for: Genetic Basis of Gluten Aggregation Properties in Wheat (Triticum aestivum L.) Dissected by QTL Mapping of GlutoPeak Parameters
Source: Front Plant Sci. 2021 Jan 28;11:611605. doi: 10.3389/fpls.2020.611605 (PMC7876098; doi:10.3389/fpls.2020.611605)

Supplementary Material

**Figure S1.** Curve of a sample produced by GlutoPeak during a test. See footnote of Table 1 for abbreviations.

**Figure S2.** Differences in GlutoPeak parameters between Luozhen No.1 and Zhengyumai 9987 tested under four environments. **p* < 0.05, ***p* < 0.01; See footnote of Table 1 for abbreviations.

**Figure S3.** Correlations between different GlutoPeak parameters. **p* < 0.05, ***p* < 0.01, ns = no significant difference; See footnote of Table 1 for abbreviations.

**Figure S4.** QTL scanning results using the GlutoPeak parameters of the RIL population tested under four environments. See footnote of Table 1 for abbreviations.

**Figure S5.** Expression level of high-confidence genes in the QTL cluster on chromosome 1DS in different wheat tissues. The heat map was plotted using the transcripts per kilobase million (TPM) values obtained from the wheat expression browser.

**Figure S6.** RP-HPLC analysis of Luozhen No. 1 and Zhengyumai 9987 on wheat glutenin.

**Figure S7.** KASP markers genotyping map based on SNP for two major QTL clusters on chromosome

1D. (A) KASP marker for 1DS QTL cluster. (B) KASP marker for 1DL QTL cluster.

**Figure S1:**


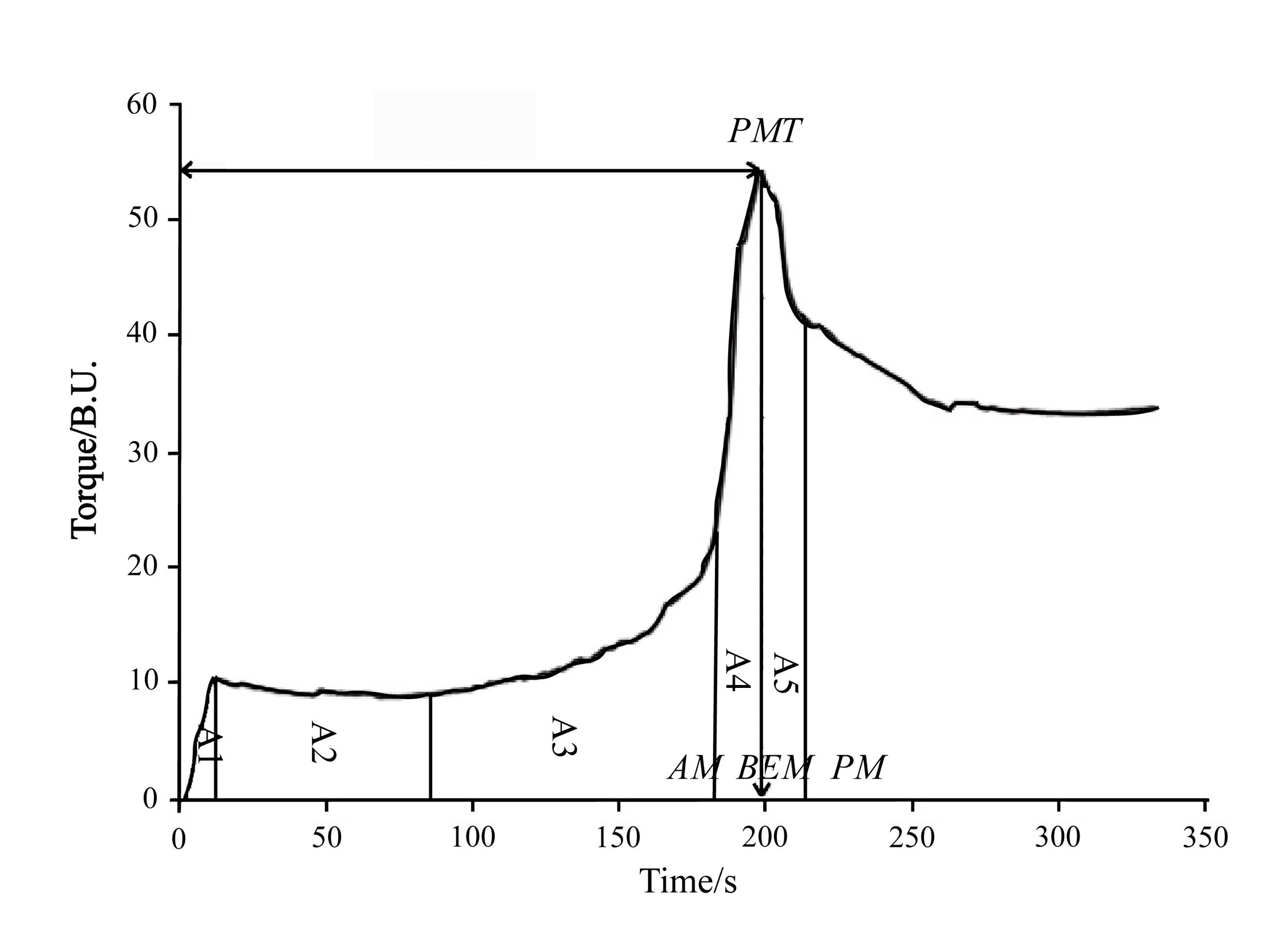


**Figure S2:**


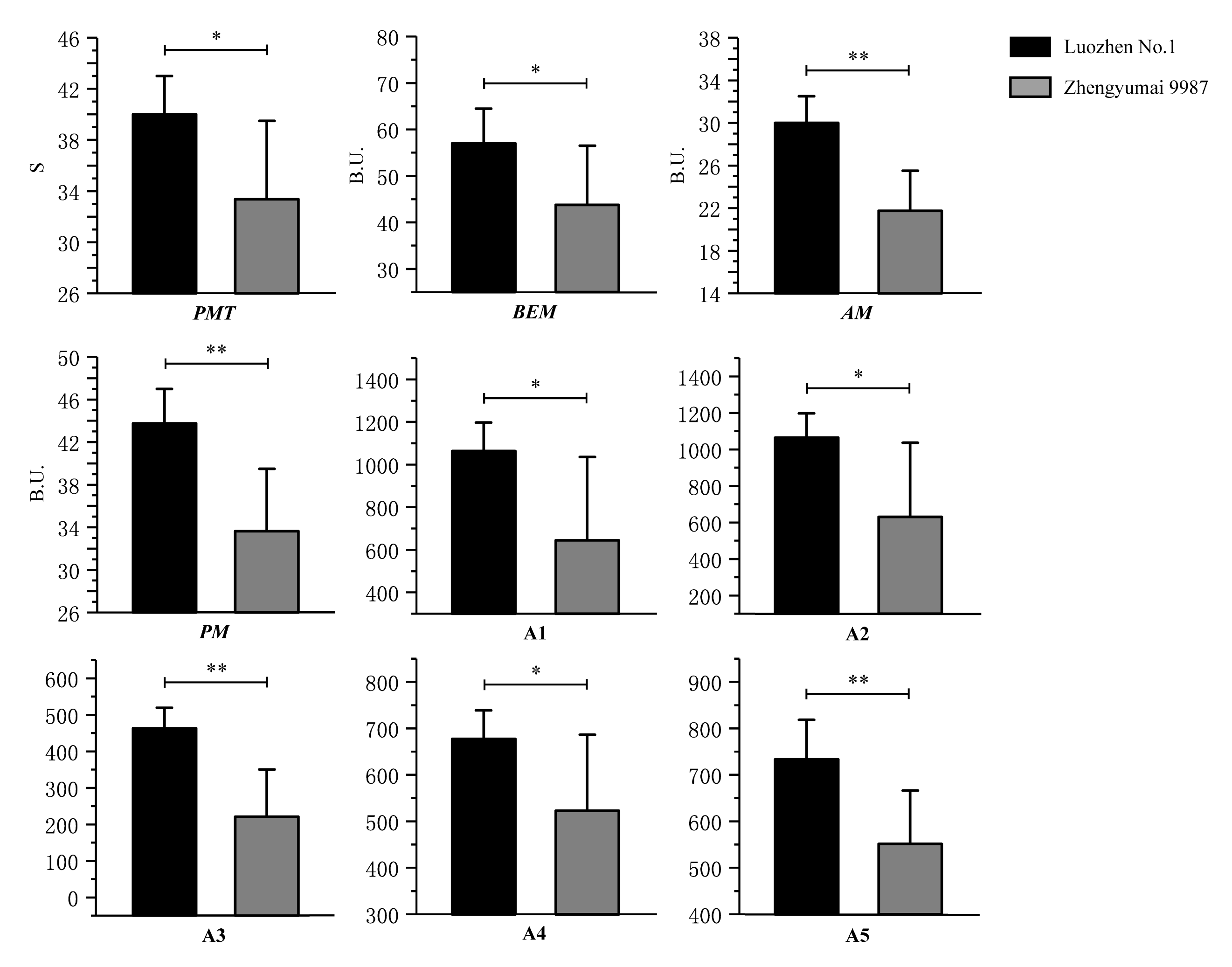


**Figure S3:**


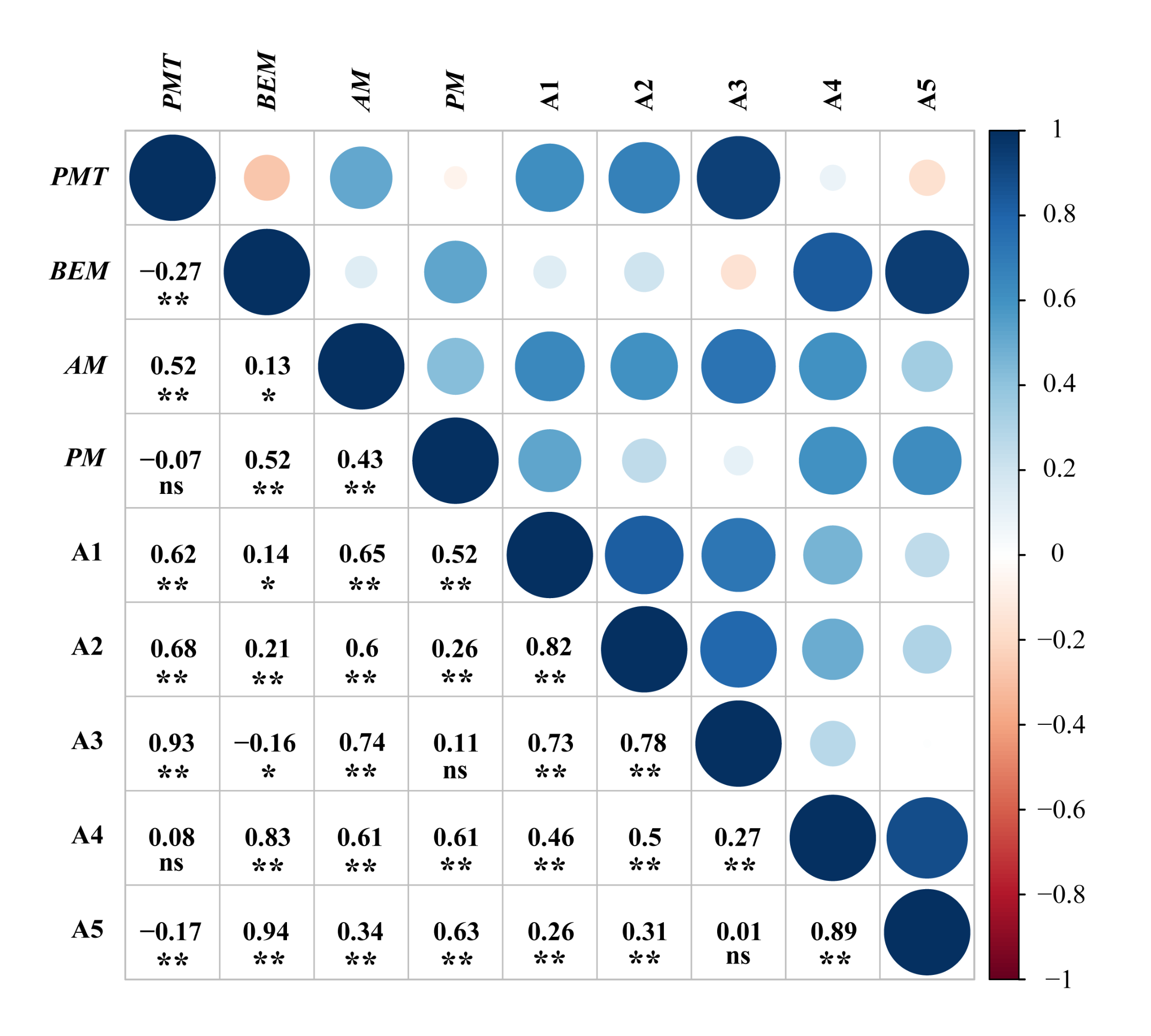


**Figure S4:**


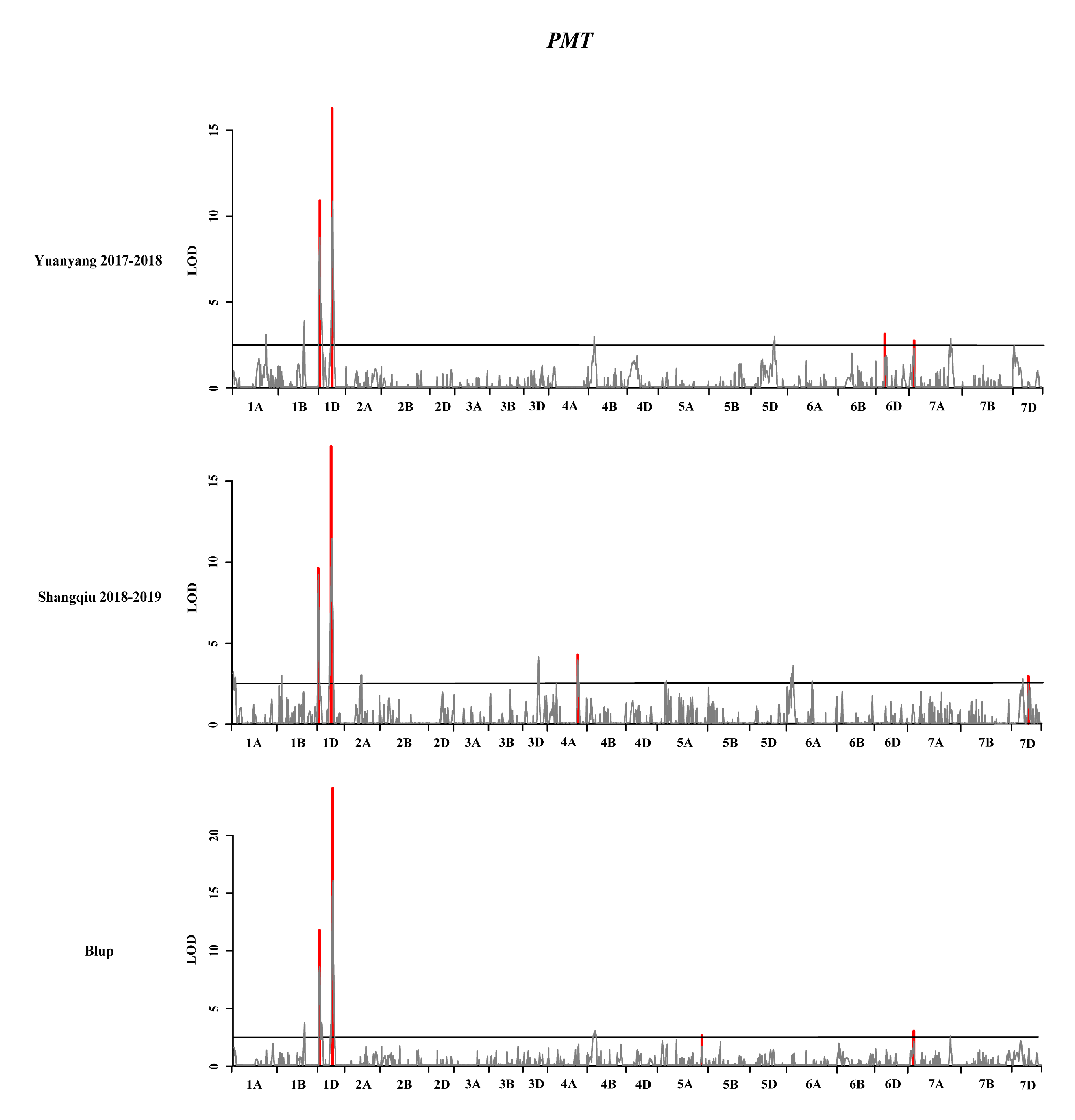


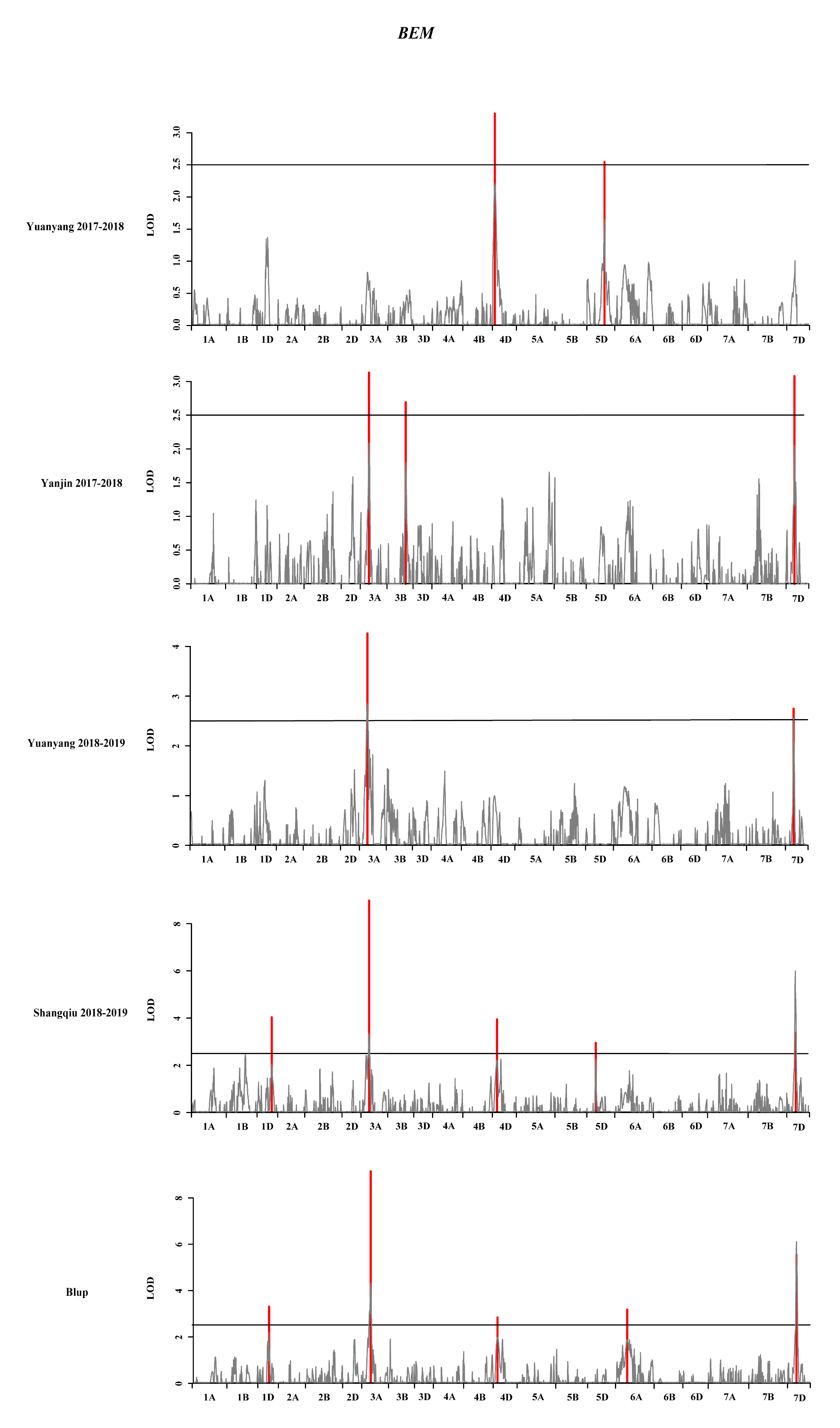


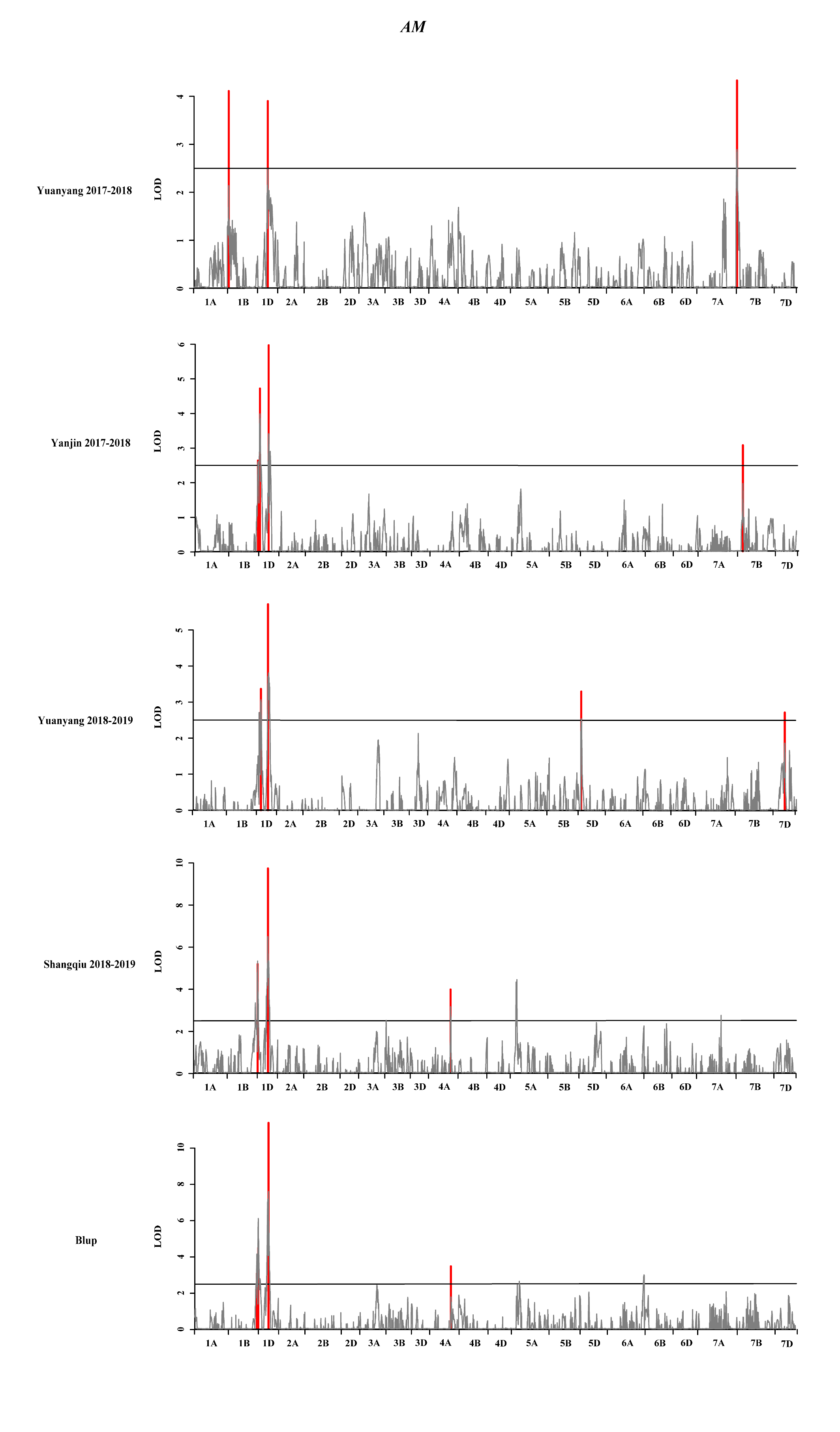


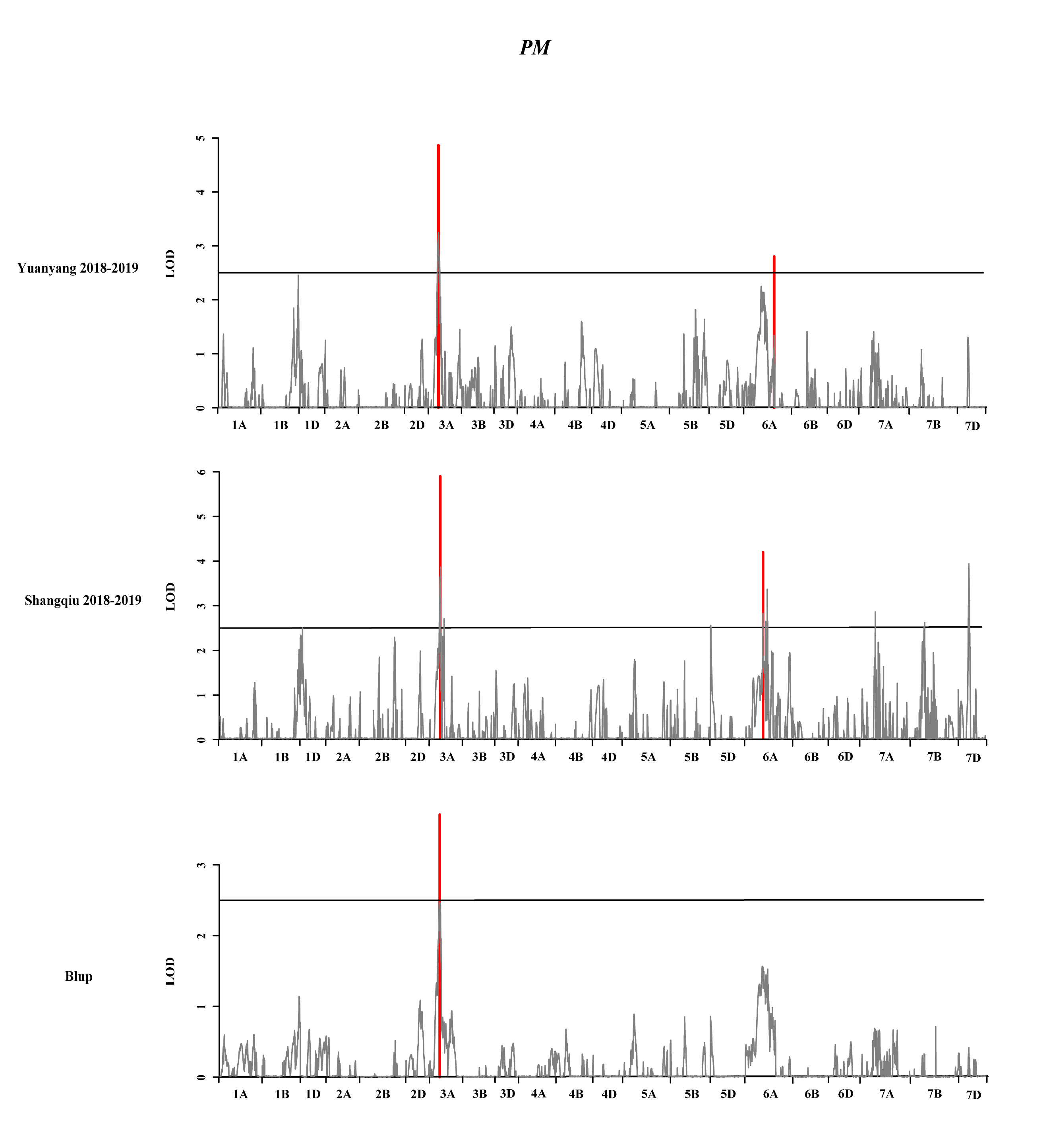


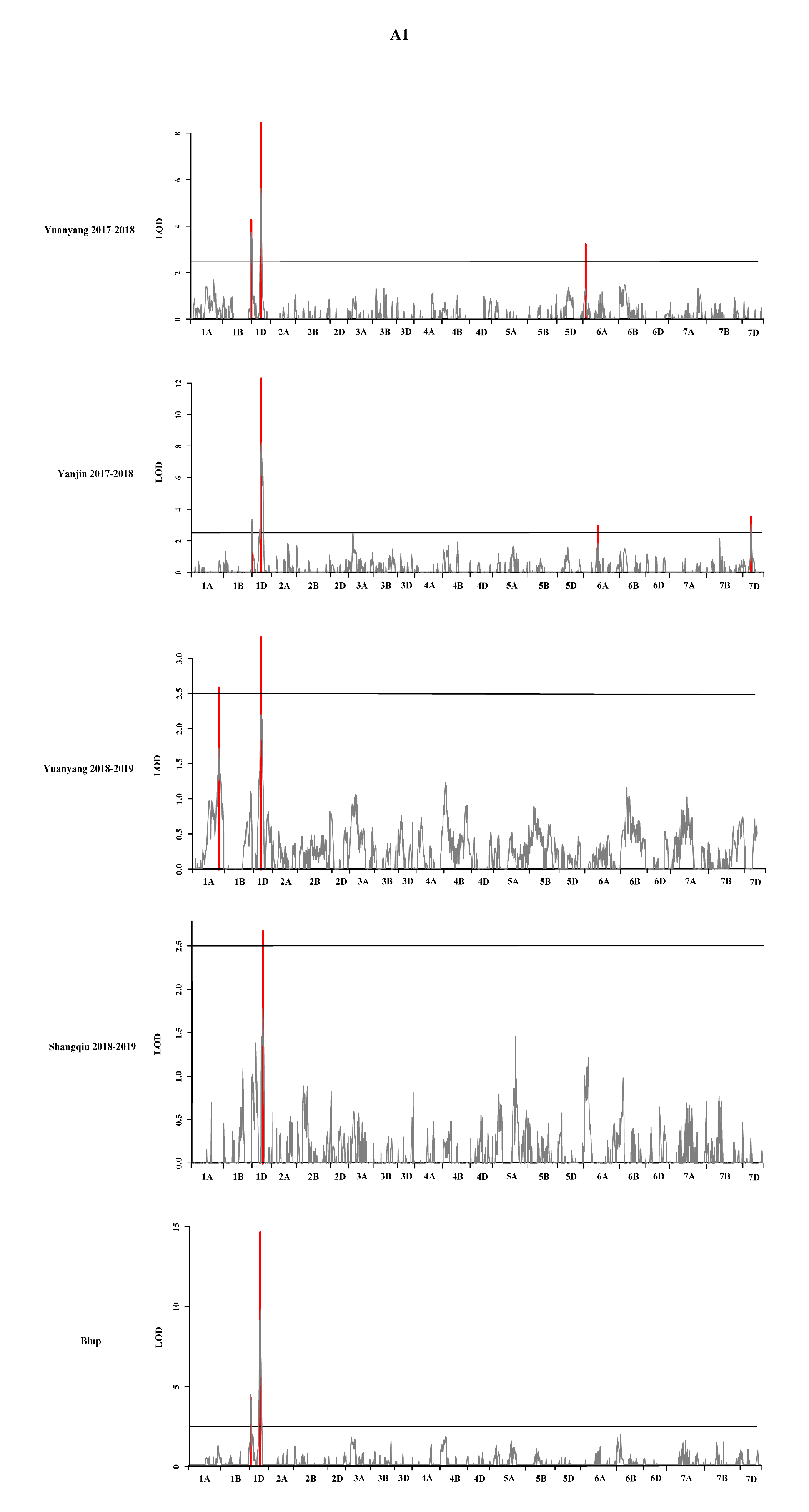


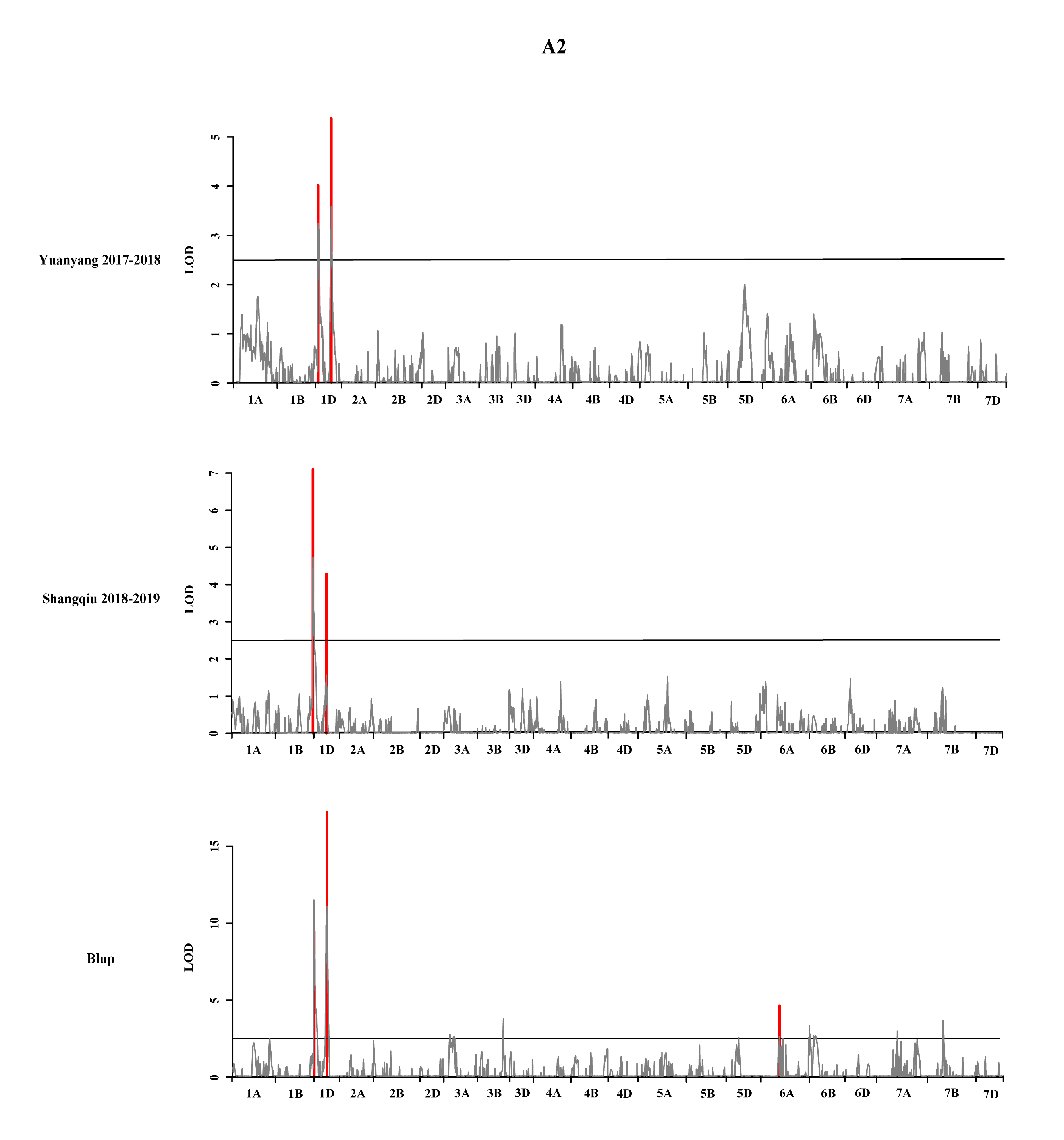


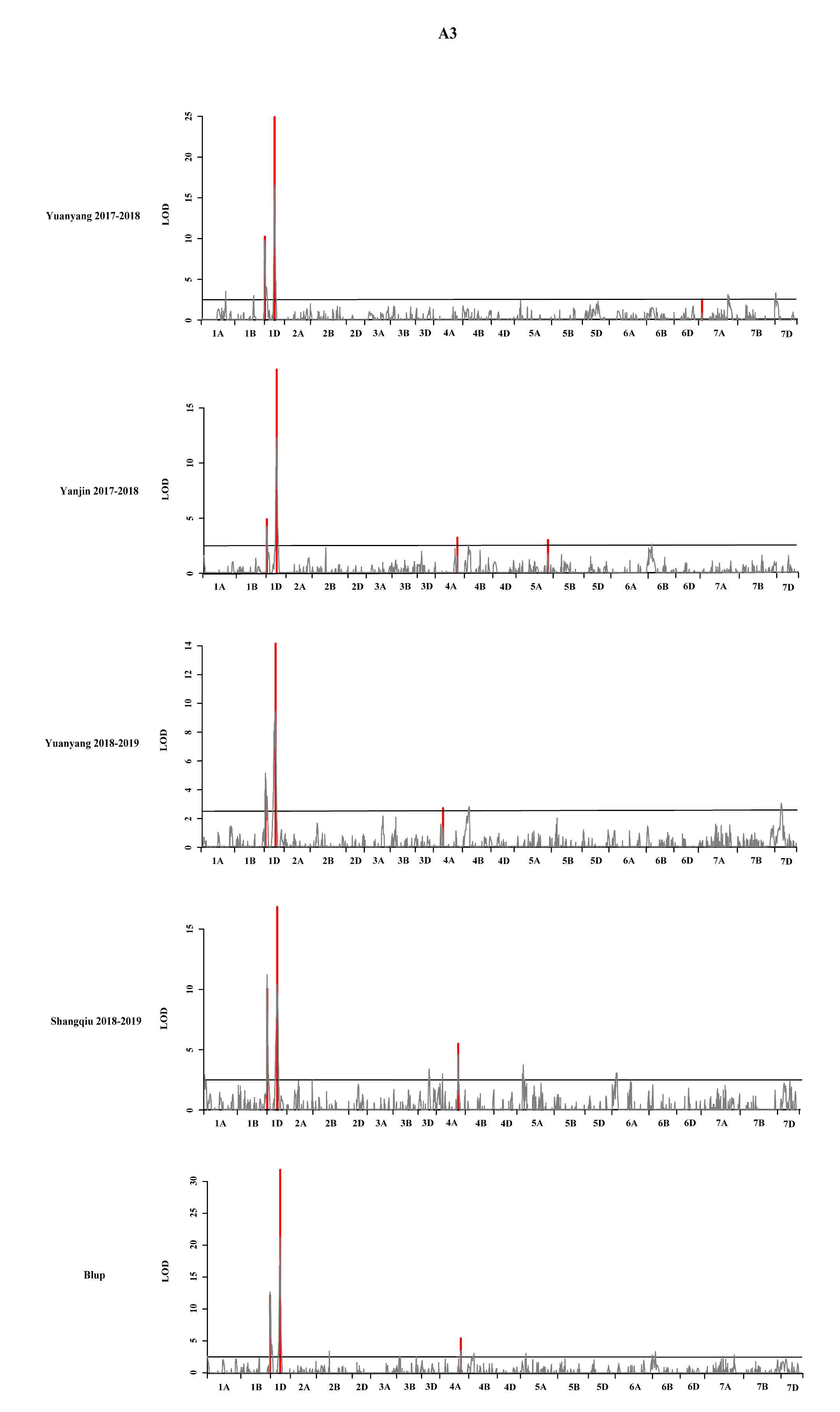


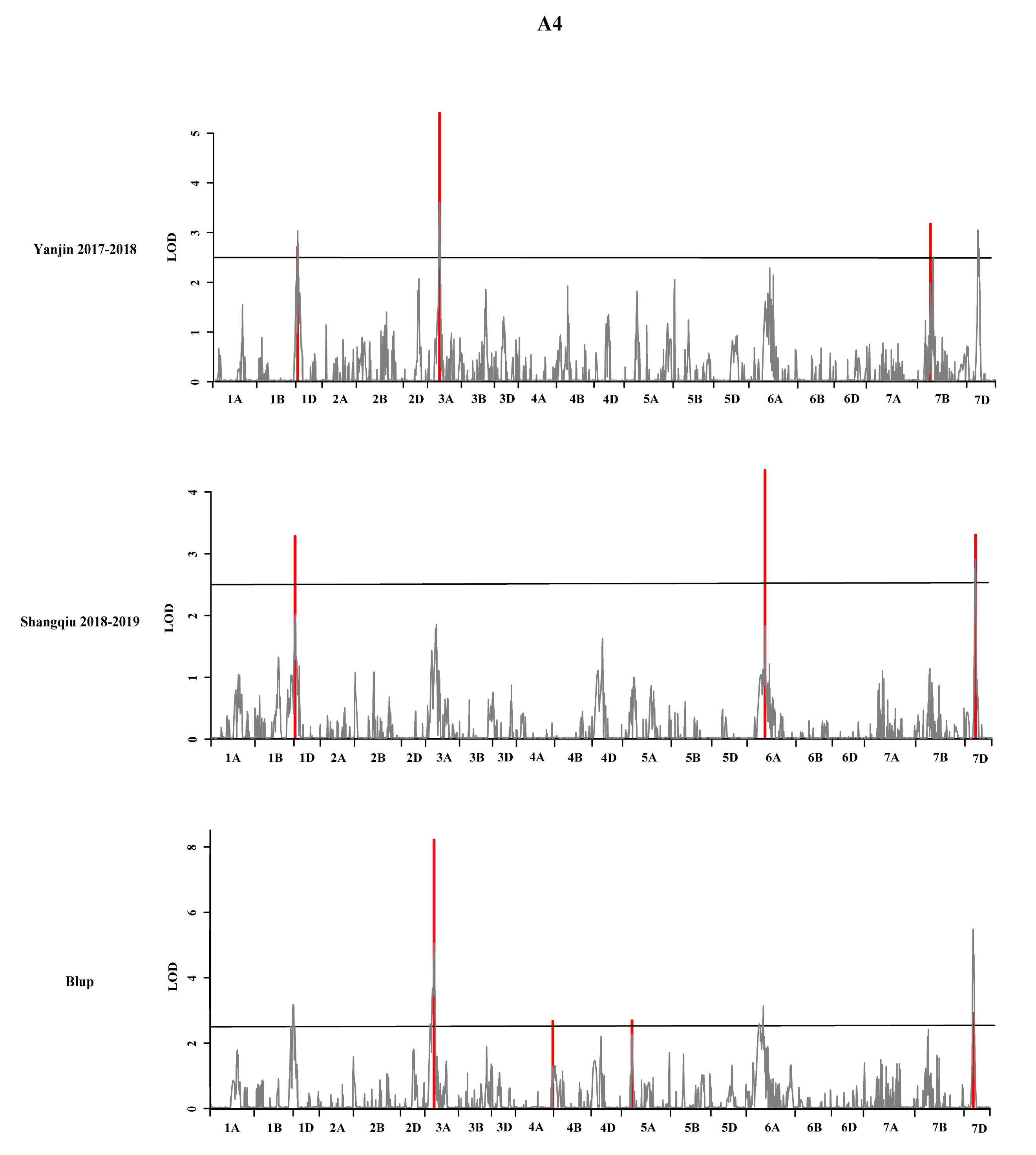


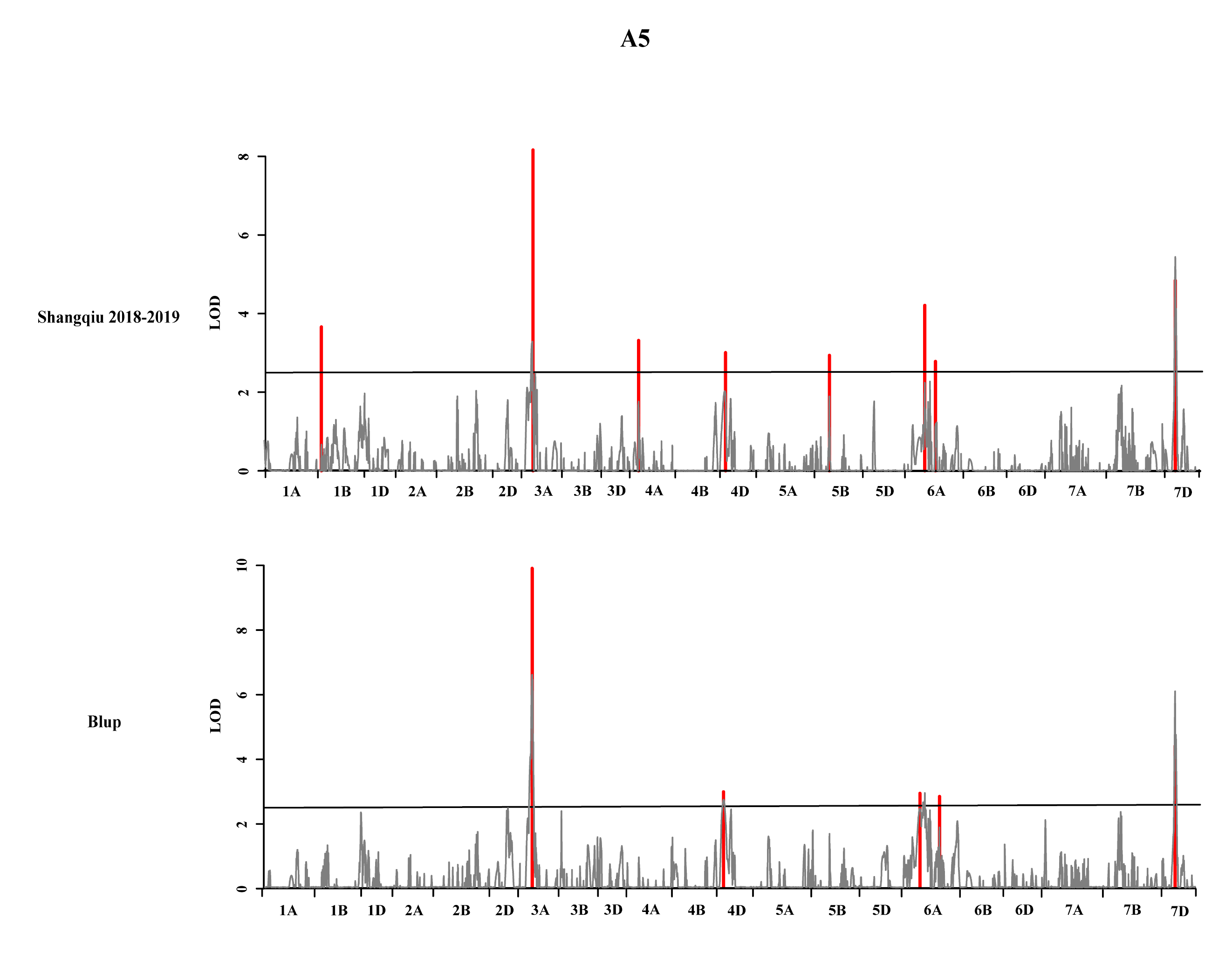


**Figure S5:**


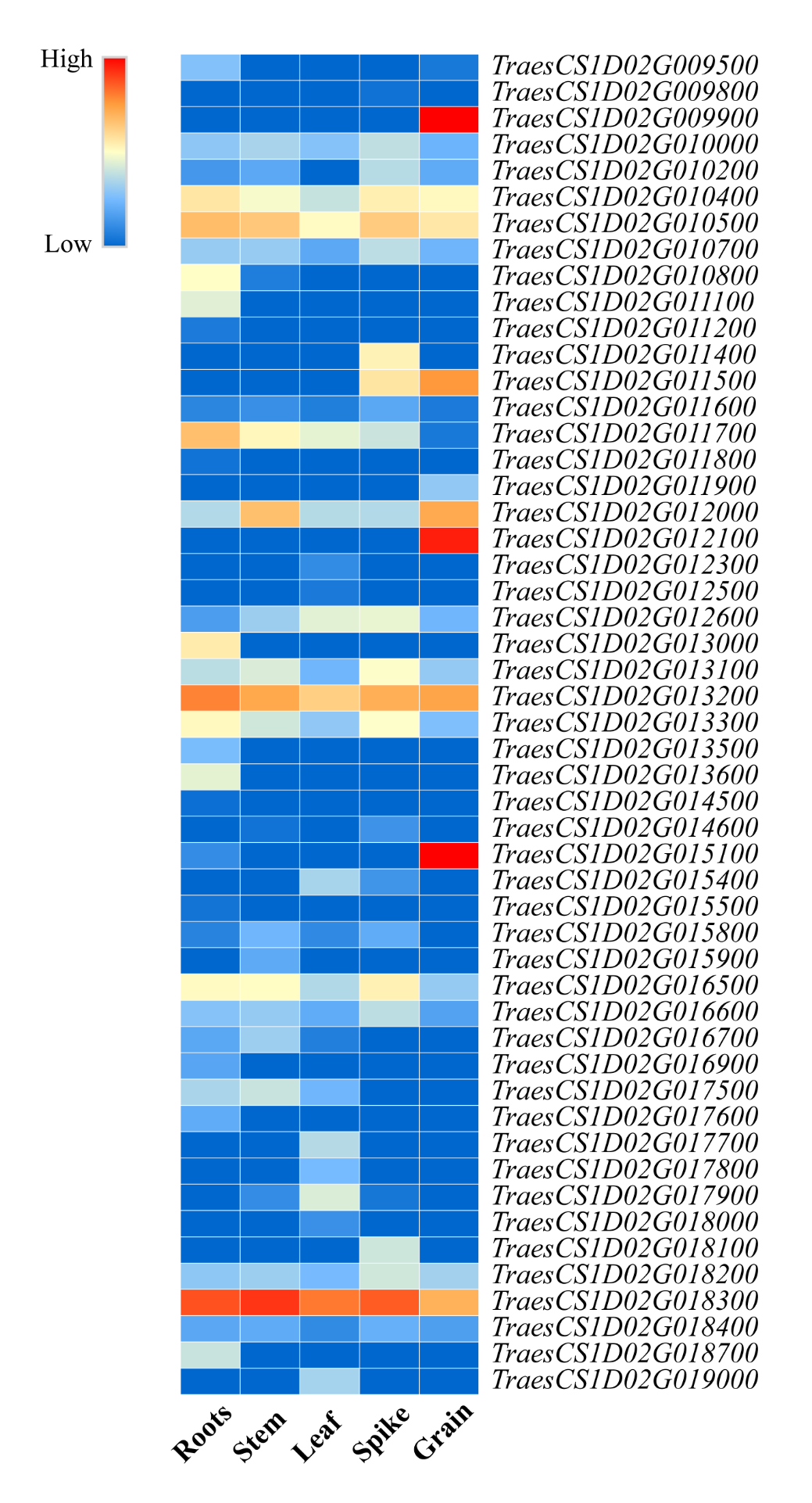


**Figure S6:**


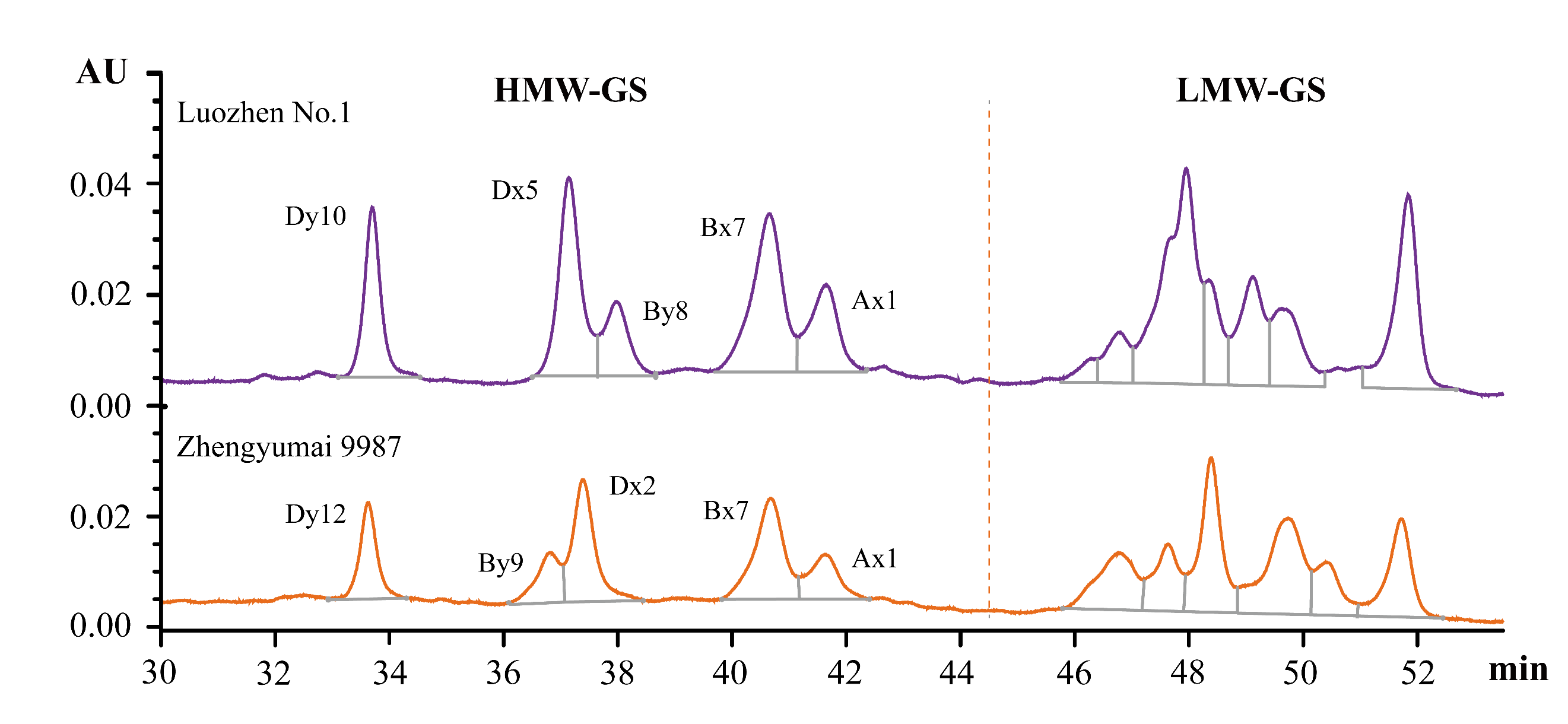


]

**Figure S7:**


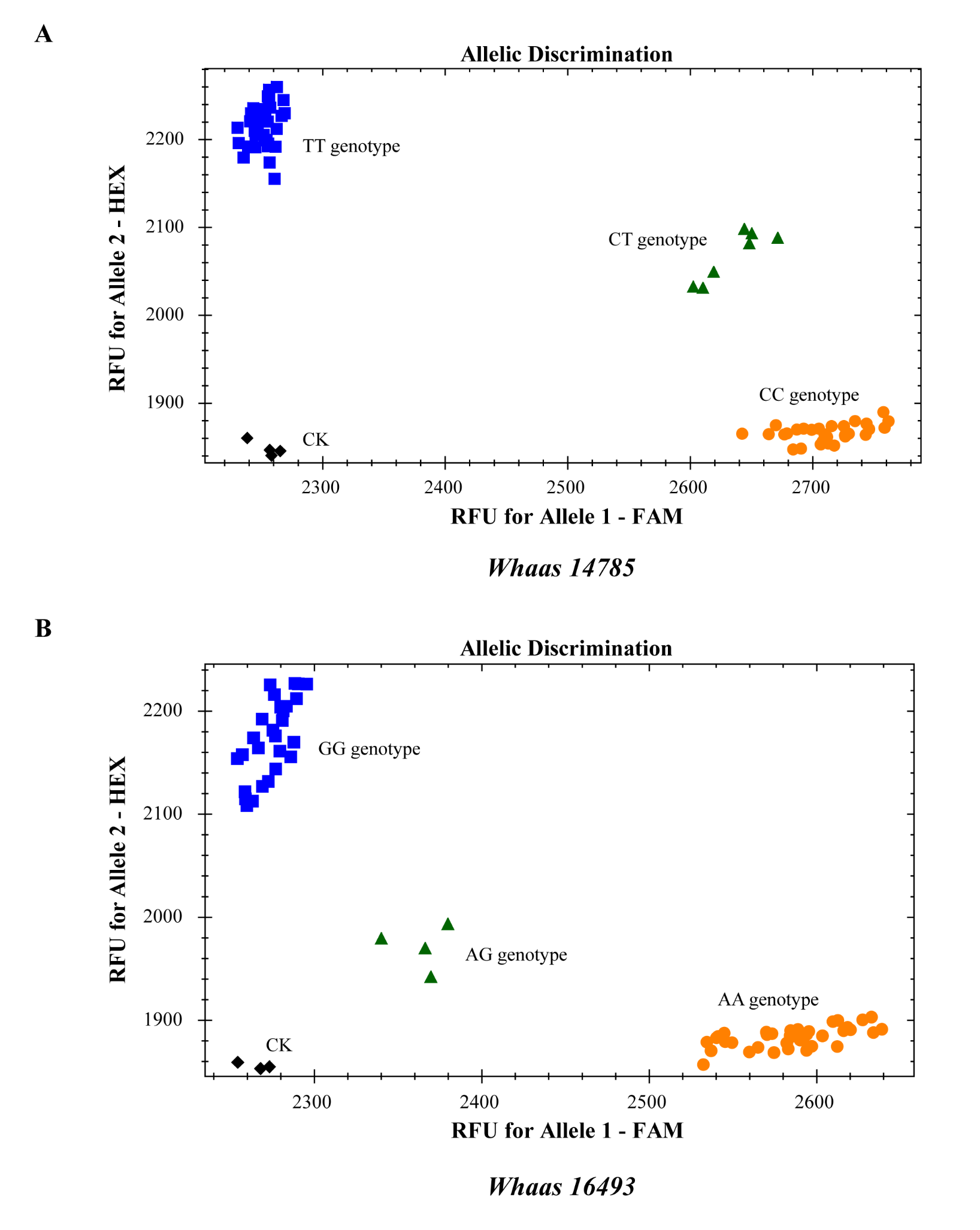

Supplement: Supplementary file 1 [file Data_Sheet_1.docx]
